# Design of an HIV Env antigen that binds with high affinity to antibodies against linear, conformational and broadly neutralizing epitopes within V1/V2

**DOI:** 10.1186/1742-4690-9-S2-O31

**Published:** 2012-09-13

**Authors:** L Liao, M Bonsignori, K Hwang, AM Moody, R Park, S Crawford, H Chen, TL Jeffries, M Cooper, X Lu, R De, N Karasavvas, S Rerks-Ngarm, S Nitayaphan, J Kaewkungwal, S Tovanabutra, P Pitisuttithum, J Tartaglia, F Sinangil, J Kim, NL Michael, GD Tomaras, Z Yang, K Dai, M Pancera, GJ Nabel, JR Mascola, PD Kwong, A Pinter, S Zolla-Pazner, MS Alam, BF Haynes

**Affiliations:** 1 Duke University Medical Center, Durham, NC, USA; 2 U.S. MHRP, Walter Reed Army Institute of Research, Silver Spring, MD, USA; 3 Department of Disease Control, Ministry of Public Health, Nonthaburi, Thailand; 4 Department of Retrovirology, US Army Medical Component, AFRIMS, Bangkok, Thailand; 5 BIOPHICS, Faculty of Tropical Medicine, Mahidol University, Bangkok, Thailand; 6 Faculty of Tropical Medicine, Mahidol University, Bangkok, Thailand; 7 Department of Research and Development, Sanofi Pasteur, Swiftwater, PA, USA; 8 Global Solutions for Infectious Diseases, South San Francisco, CA, USA; 9 Vaccine Research Center/NIH, Bethesda, MD, USA; 10 Public Health Research Institute Center, New Jersey Medical School, Newark, NJ, USA; 11 Veterans Affairs New York Harbor Healthcare System, Manhattan Campus, New York, NY, USA

## Background

The RV144 HIV-1 vaccine trial showed protection from HIV-1 acquisition with vaccine efficacy of 31.2%. Study of the immune correlates demonstrated an inverse association of V1/V2 antibodies with infection risk. A key task for HIV-1 vaccine development is to improve the level of efficacy seen in the RV144 trial with subsequent vaccine designs.

## Methods

E.A244 V1/V2 Env tags contains an N-terminal Ig leader sequence and C-terminal Avi- and His6-tags linked to the V1/V2 domain, was expressed in 293F cells and purified by nickel column. Binding of Tier 1 neutralizing mAb CH58 from RV144 vaccinees, V2 conformational mAb 697D and broadly neutralizing antibodies (bnAb) CH01 and PG9/PG16 to 33 HIV-1 gp140/gp120s and 12 HIV-1 V1/V2 scaffold Envs was tested by ELISA and surface plasmon resonance.

## Results

Among 45 HIV-1/SIV Envs tested, E.A244 V1/V2 tags and E.A244 gp120Δ11 Env were the only Env antigens recognized by all three types of mAbs: CH58, 697D, and bnAbs CH01, and PG9/PG16. E.A244 V1/V2 tag bound CH58 with a Kd of 0.33 nM and 697D with a Kd of 117 nM. Although PG9 preferentially recognizes trimers, PG9 bound well to both E.A244 gp120Δ11 (Kd = 47.3) and E.A244 V1/V2 tags (Kd = 83.3 nM). BnAb CH01 bound V1/V2 tags as well (Kd = 334 nM). E.A244 V1/V2 Env tags was also recognized by the unmutated ancestor antibodies (UAs) of CH58 with ELISA EC50 = 4.9 nM and CH01 with EC50 = ~1μM. E.A244 V1/V2 tags and AE.gp70 V1/V2 scaffold were the best recombinant Envs for detection of plasma V1/V2 antibodies in RV144 vaccinees.

## Conclusion

Recombinant E.A244 V1/V2 Env tags Env expresses linear as well as conformational determinants recognized by V1/V2 mAbs and some of their UAs. This V1/V2 construct is a candidate immunogen to target RUAs and intermediate ancestors of V1/V2 antibodies to drive their induction.

